# Supplementary material for: Programming T cells for intercellular genome editing
Source: bioRxiv. 2026 Jun 23:2026.06.21.729417. Preprint. [Version 1] doi: 10.64898/2026.06.21.729417 (PMC13320803; doi:10.64898/2026.06.21.729417)
Supplement: Supplement 1 [file NIHPP2026.06.21.729417v1-supplement-1.pdf]

bioRxiv preprint doi: https://doi.org/10.64898/2026.06.21.729417; this version posted June 23, 2026. The copyright holder for this preprint (which was not certified by peer review) is the author/funder, who has granted bioRxiv a license to display the preprint in perpetuity. It is made available under a CC-BY 4.0 International license.

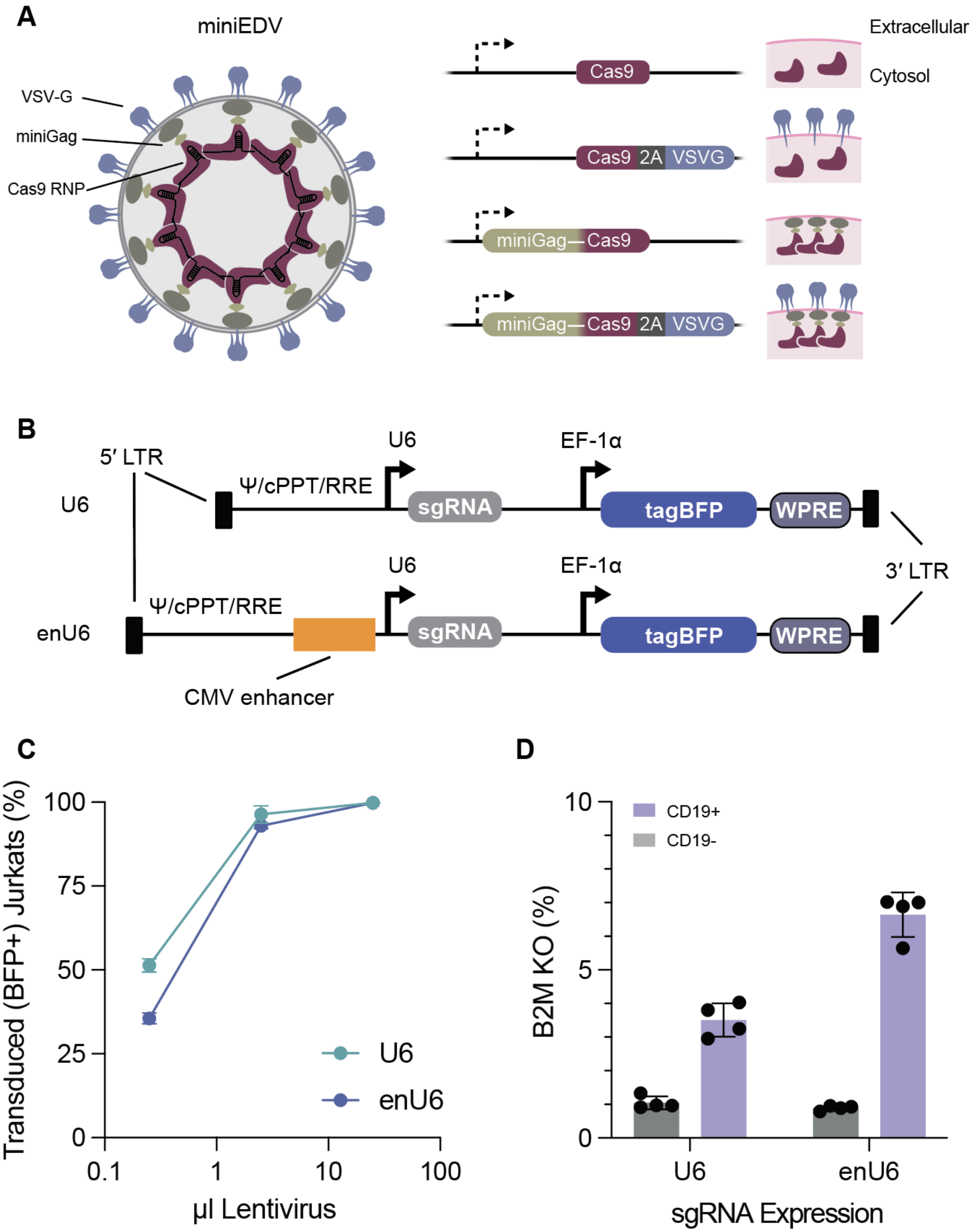

Supplementary Figure 1:

bioRxiv preprint doi: https://doi.org/10.64898/2026.06.21.729417; this version posted June 23, 2026. The copyright holder for this preprint (which was not certified by peer review) is the author/funder, who has granted bioRxiv a license to display the preprint in perpetuity. It is made available under a CC-BY 4.0 International license.

A. Cartoon of miniGag EDV structure showing VSV-G fusogen (blue), miniGag (green), and Cas9 RNP (red). Right: Constructs used in Figure 1 with expected outcomes in producer cells at the plasmid membrane.
B. Schematic of lentiviral vectors used to install *B2M*-targeted sgRNA expression in producer and target cells, using either U6 alone (top) or U6 downstream of the CMV enhancer (bottom).
C. Transduction (BFP^+) in Jurkat producer cells quantified by flow cytometry following volume-based transductions with either of the constructs in (B). (*n* = 4 biological replicates)
D. *B2M* editing in target cells following co-culture with Jurkat producer cells transduced with the 50µl dose of lentivirus from (C). (*n* = 4 biological replicates)

bioRxiv preprint doi: https://doi.org/10.64898/2026.06.21.729417; this version posted June 23, 2026. The copyright holder for this preprint (which was not certified by peer review) is the author/funder, who has granted bioRxiv a license to display the preprint in perpetuity. It is made available under a CC-BY 4.0 International license.

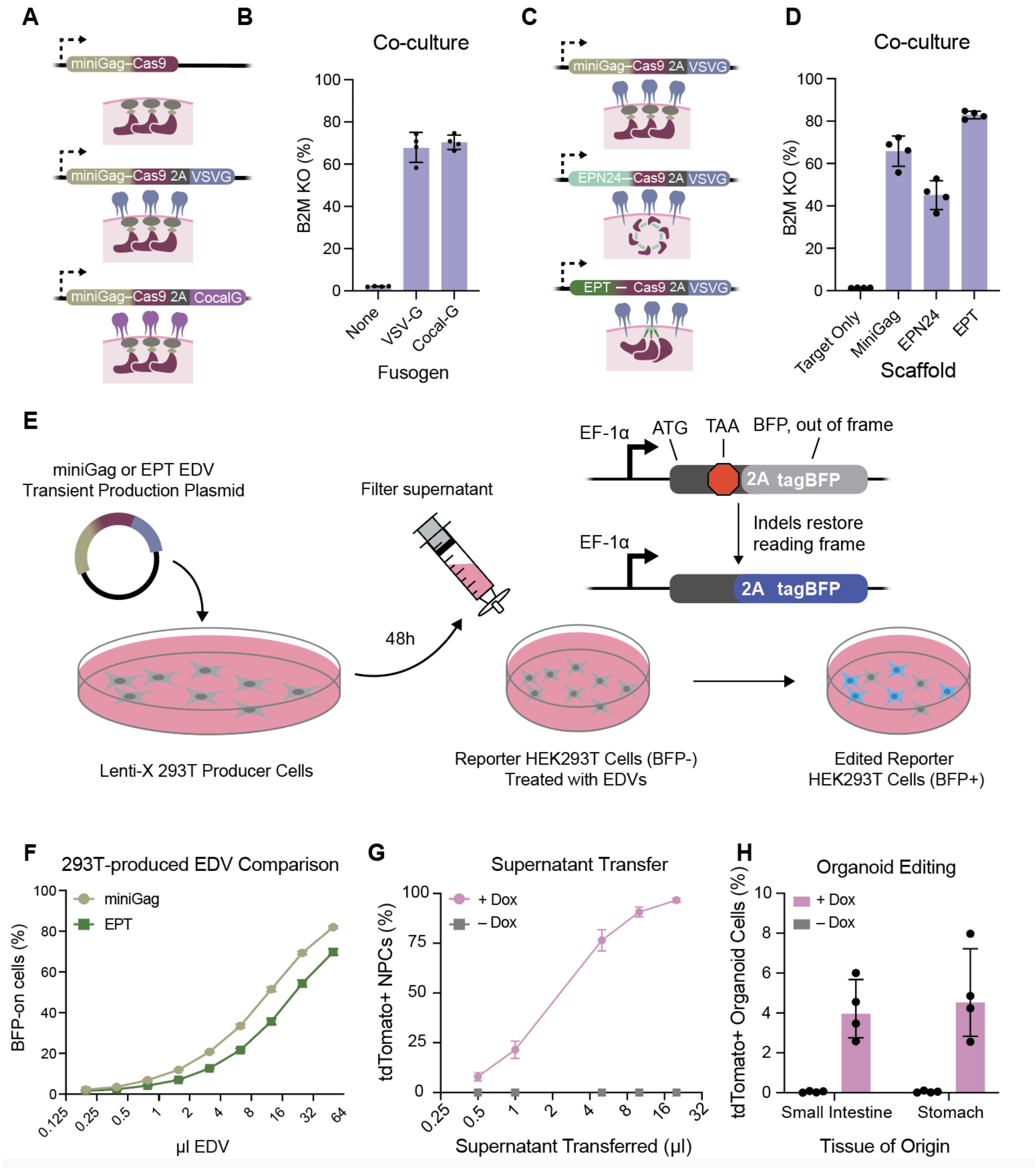

Supplementary Figure 2:

A. Visual representations of EDV cassettes encoding for no fusogen, VSV-G, or Cocal-G.

B. Equivalent target cell editing using Jurkats expressing miniGag-Cas9 EDVs pseudotyped with VSV-G or Cocal-G measured by flow cytometry following 6 days of co-culture. (*n* = 4 biological replicates)

C. MiniGag, EPN24, and EPT EDV cassette visual representations.

bioRxiv preprint doi: https://doi.org/10.64898/2026.06.21.729417; this version posted June 23, 2026. The copyright holder for this preprint (which was not certified by peer review) is the author/funder, who has granted bioRxiv a license to display the preprint in perpetuity. It is made available under a CC-BY 4.0 International license.

D. Extension of Figure 2B including EPN24; Loss of β2M measured by flow cytometry indicates EPN24-based EDV construct leads to less efficient Cas9 transfer following co-culture in the presence of doxycycline. (n = 4 biological replicates)
E. Schematic of Cas9 editing reporter assay. Plasmids encoding miniGag- or EPT-based EDVs driven by the CAG promoter and a U6-sgRNA cassette were transfected into Lenti-X 293T cells. EDVs were harvested and filtered from supernatant and used to treat HEK293T targets with genomically integrated indel reporter cassettes, in which a premature stop codon prevents BFP expression until Cas9-mediated indels restore the BFP reading frame. Editing is quantified as BFP expression by flow cytometry.
F. Comparison of editing results after treatment with miniGag or EPT- based Cas9 EDVs produced by Lenti-X 293T cells. (n = 3 biological replicates)
G. Jurkat cells were cultured in doxycycline to produce EPT-Cre EDVs. Supernatant was filtered and added to Ai9 NPCs. tdTomato fluorescence in NPCs indicates successful delivery of Cre. (n = 4 biological replicates)
H. Flow cytometry quantification of Cre delivery to organoids from Ai9 mouse stomach and small intestines following co-culture with producer Jurkats. (n = 4 biological replicates)

bioRxiv preprint doi: https://doi.org/10.64898/2026.06.21.729417; this version posted June 23, 2026. The copyright holder for this preprint (which was not certified by peer review) is the author/funder, who has granted bioRxiv a license to display the preprint in perpetuity. It is made available under a CC-BY 4.0 International license.

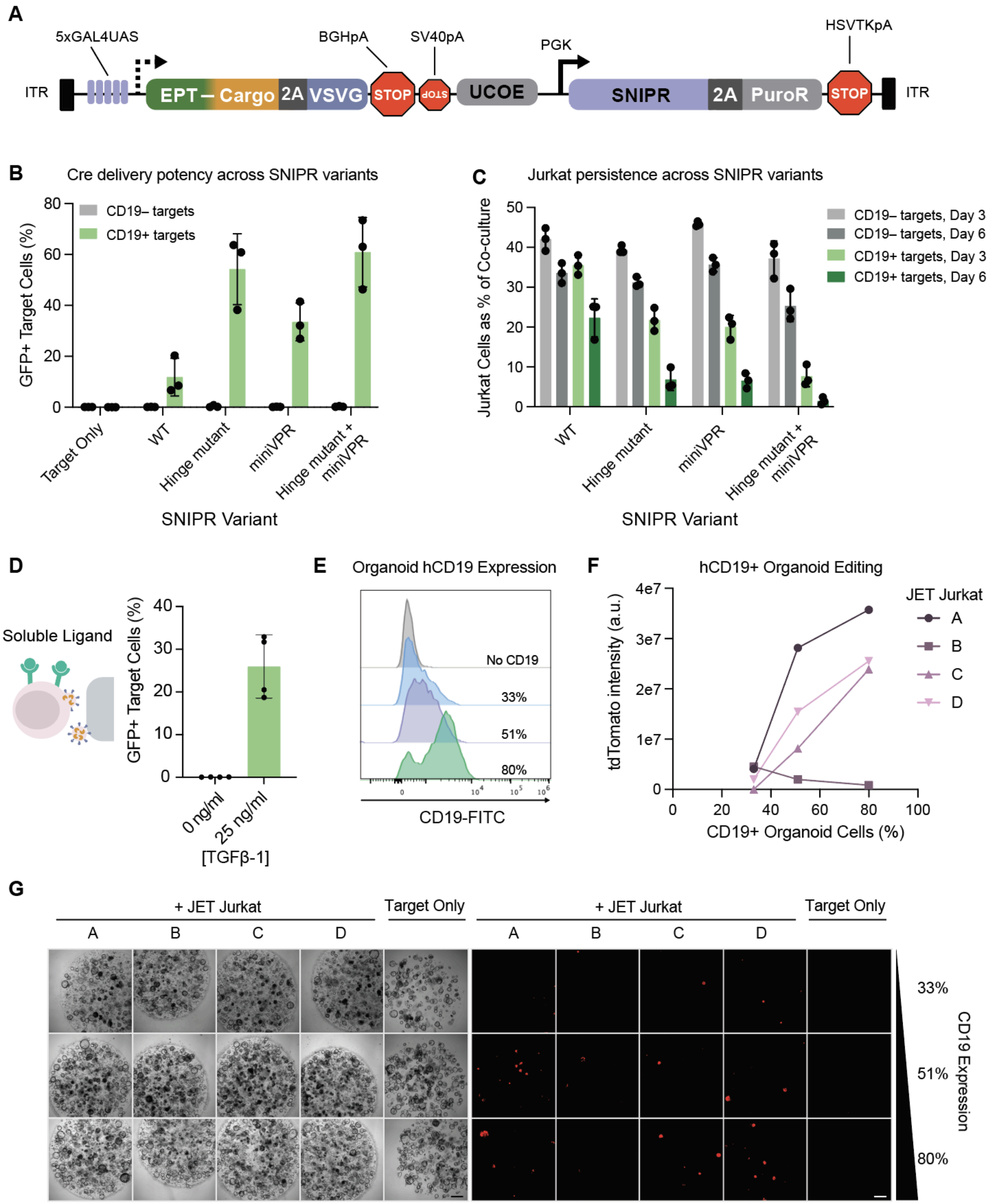

Supplementary Figure 3:

A. Map of all-in-one JET Sleeping Beauty construct. 5xGAL4UAS + minimal promoter drives inducible EDV expression. SNIPR and puromycin resistance are driven by a PGK promoter with ubiquitous chromatin opening element (UCOE). As the UCOE is

bioRxiv preprint doi: https://doi.org/10.64898/2026.06.21.729417; this version posted June 23, 2026. The copyright holder for this preprint (which was not certified by peer review) is the author/funder, who has granted bioRxiv a license to display the preprint in perpetuity. It is made available under a CC-BY 4.0 International license.

essentially a bidirectional promoter, an inverted SV40 poly(A) sequence was included to terminate antisense transcription[40].

B. JET Jurkats were generated with previously described SNIPR variants. GFP fluorescence in target cells after 3 days of co-culture is shown for each condition. (P:T = 1, *n* = 3 biological replicates)

C. Quantification of Jurkats as a percent of total cells in co-culture after 3 and 6 days after initially being seeded at 50%, using the SNIPR variants tested in (B). (*n* = 3 biological replicates)

D. Cre delivery assessed by flow cytometry measurement of GFP expression in target *lsl*-GFP HEK293T cells following 3 days of co-culture with JET Jurkat cells (anti-TGFβ) in the absence or presence of 25 ng/ml TGFβ1. (*n* = 4 biological replicates)

E. Human CD19 expression on engineered shAPC, *Kras*^{G12D};*Trp53*$^{-/-}$ mutant (AKP) colon cancer organoid lines.

F. Quantification of fluorescent intensities from co-cultures with various JET Jurkat producers with organoids expressing hCD19 at various levels. Each point represents the average of technical replicates for a given Jurkat biological replicate. Lack of function in JET Jurkat line B is likely due to transgene silencing, resulting in reduction in transfer efficiency as engineered producer cells are kept in culture over time[41].

G. Brightfield (left) and red fluorescence (right) representative images of *lsl*-tdTomato hCD19+ organoids after four days of co-culture with CD19-responsive JET Jurkat cells, related to (F). (scale bar = 500 μm)

bioRxiv preprint doi: https://doi.org/10.64898/2026.06.21.729417; this version posted June 23, 2026. The copyright holder for this preprint (which was not certified by peer review) is the author/funder, who has granted bioRxiv a license to display the preprint in perpetuity. It is made available under a CC-BY 4.0 International license.

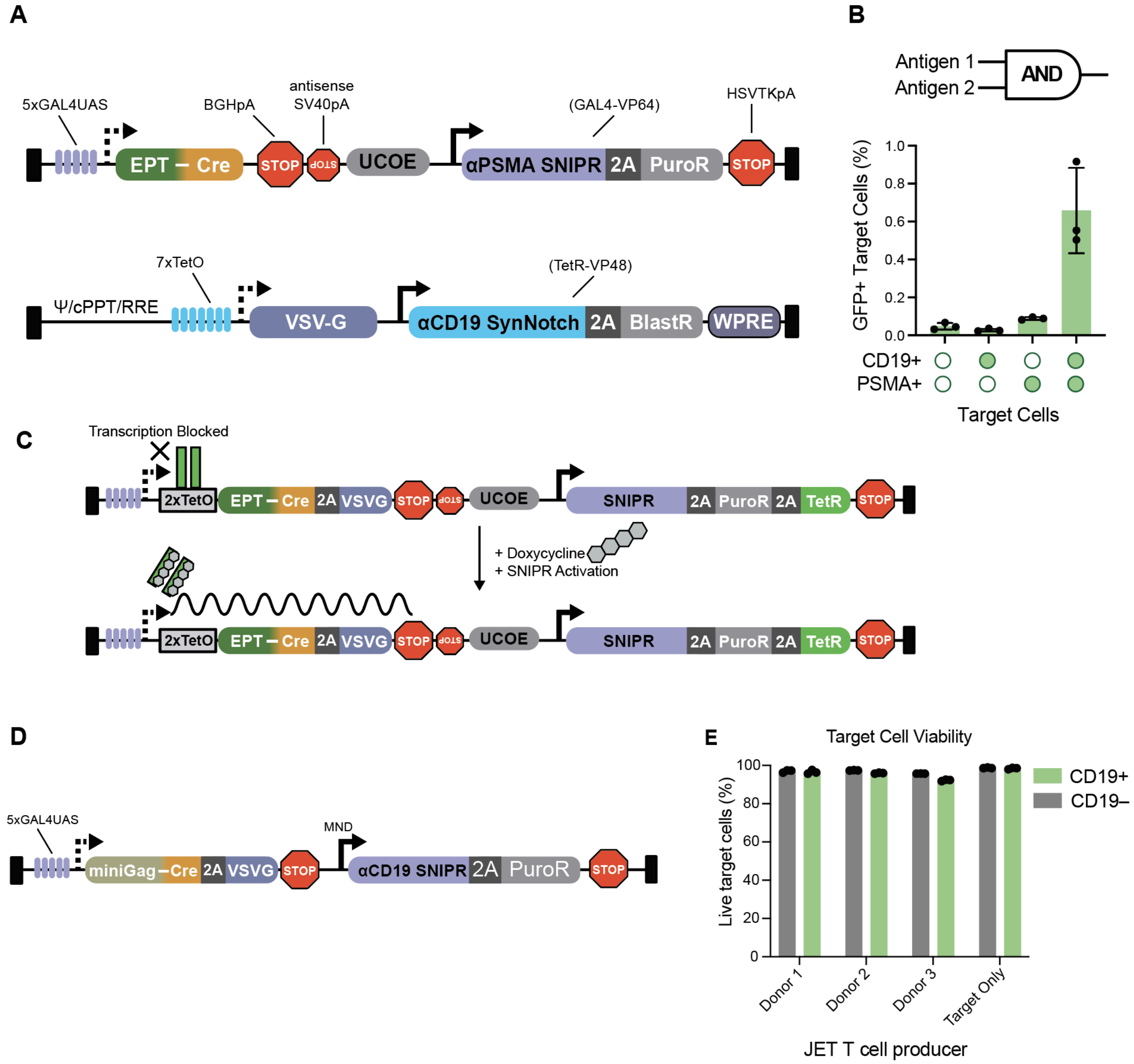

Supplementary Figure 4:

A. Schematics of dual-ligand AND-gated constructs.

B. Flow cytometry measurement of GFP expression in target HEK293T cells expressing CD19, PSMA, both, or neither following 3 days of co-culture with AND-gated JET Jurkat cells.

C. Schematics of ligand+doxycycline AND gated construct. (Related to Fig. 4C)

D. Schematic of Sleeping Beauty construct used for primary JET T cell generation.

E. Viability data for target cells following 3 days of co-culture with primary T cells. (Related to Figure 4G)
